# Transparent qubit manipulations with spin-orbit coupled two-electron nanowire quantum dot

**DOI:** 10.1038/s41598-021-98152-z

**Published:** 2021-09-22

**Authors:** Kuo Hai, Yifan Wang, Qiong Chen, Wenhua Hai

**Affiliations:** Department of Physics and Key Laboratory of Low Dimensional Quantum Structures and Quantum Control of Ministry of Education, Hunan Normal University, Changsha, 410081 China

**Keywords:** Quantum information, Computational science

## Abstract

We report on the first set of exact orthonormalized states to an ac driven one-dimensional (1D) two-electron nanowire quantum dot with the Rashba–Dresselhaus coexisted spin-orbit coupling (SOC) and the controlled magnetic field orientation and trapping frequency. In the ground state case, it is shown that the spatiotemporal evolutions of probability densities occupying internal spin states and the transfer rates between different spin states can be adjusted by the ac electric field and the intensities of SOC and magnetic field. Effects of the system parameters and initial-state-dependent constants on the mean entanglement are revealed, where the approximately maximal entanglement associated with the stronger SOC and its insensitivity to the initial and parametric perturbations are demonstrated numerically. A novel resonance transition mechanism is found, in which the ladder-like time-evolution process of expected energy and the transition time between two arbitrary exact states are controlled by the ac field strength. Using such maximally entangled exact states to encode qubits can render the qubit control more transparent and robust. The results could be extended to 2D case and to an array of two-electron quantum dots with weak neighboring coupling for quantum information processing.

Coherent manipulation of electron spin is one of the central problems of spintronics and is critically important to quantum computing and information processing with spins^1–4^. The orbital part of the spin-orbit entangled states of a charged particle can be used for the qubit manipulation^5–8^, in the presence of ac electric field and static magnetic field^3,9–13^. The previous investigation has paved the way for individually manipulating electron spins in a locally gated few-quantum-dot system^11,14–16^ or an array of quantum dots^17–19^. There were great theoretical and experimental efforts for researching the semiconducting nanowire quantum dots (NQDs) with spin-orbit coupling (SOC)^20–28^, because of their potential utility to topologically fault-tolerant quantum computation^29–32^. The Rashba and Dresselhaus terms of SOC can be transformed into each other under a spin rotation^33,34^ and also can be tunable by using a periodic field^35,36^. A qubit refers to a two-level system^37^, and the qubit manipulation is necessary for realizing the quantum gate between qubits. *Two-qubit gate is practically important in any scheme of universal quantum computing*^5,6,38–40^.

The exact analytical solutions to the time-dependent Schrödinger equation describing a driven two-level system are invaluable in the context of qubit control^41,42^. An obvious advantage is that the exact results can render the control strategies more transparent^43^. The charged two-particle problem was widely investigated and the exact solutions were constructed analytically for the different confinements^44–47^. A lot of generalized coherent states of harmonic systems was derived^48–50^, which describes some orthonormalized complete sets of Schrödinger cat states^51,52^. Such cat states have been experimentally prepared as the spin-motion entangled states of a trapped ion^6,53^. The photonic Schrödinger cats have also been obtained exactly and been controlled well^54^. However, *it is notoriously difficult to acquire the exact analytical solutions of a SO coupled and ac driven two-electron system*.

In the present paper, we consider a pair of SO coupled and ac driven two-spin electrons confined in a one-dimensional (1D) NQD^27^. We seek a set of orthonormalized exact solutions of the system by managing the magnetic field orientation to match the SOC-dependent phase^12^ and selecting the specific trapping frequencies to fit the known exact stationary states of the relative motion^44–47^. As probability amplitudes of the exact spin states, the complete solutions of motional states are the superpositions of the generalized coherent states with superposition constants determined by the initial states. The square norm of a motional state describes the probability density occupying the corresponding spin state and behaves as a kind of oscillating wave packets. The different initial-state-dependent constant sets correspond to the different ground states with the lowest vibrational quantum number. For any ground state we show that the spatiotemporal evolutions of probability densities and the transfer rates between different spin states can be adjusted by the ac electric field and the intensities of SOC and magnetic field. Effects of the system parameters and initial constants on the mean entanglement measured by the average linear entropy are studied. It is revealed numerically that the exact ground state with the approximately maximal entanglement is associated with the stronger SOC and is insensitive to the initial and parametric perturbations. In any one of the orthonormalized states, the expected energy consists of a quantum part and a continuously time-varying one, which is used to illustrate a novel resonance transition mechanism where the transition time between two arbitrary states and the corresponding ladder-like time-evolution process of the expected energy^50^ are transparently controlled by the ac field strength. Our exact maximally entangled states can be used to encode the qubits and to render the qubit manipulation more transparent and robust. The results could be extended to a 2D quantum-dot-electron system^28^ and could be applied to quantum information processing with an array of electron pairs separated from each other by different quantum dots with weak neighboring coupling as perturbation^2^.

## Results and discussions

### Exact and orthonormalized complete solutions

We consider a gated NQD with the Rashba–Dresselhaus coexisted SOC, where a pair of two-spin electrons is confined in a 1D harmonic trap controlled by the voltages on the static electric gates, and subject to an arbitrarily strong ac electric field and static magnetic field. The two-qubit system is governed by the effective Hamiltonian^12,27^

1 $$\begin{aligned} H& = H_0+H_{\sigma }, \nonumber \\ H_0& = \sum _{k=1}^2\Big [-\frac{p_k^2}{2m_{eff}}+\frac{1}{2} m_{eff}\omega ^2 x_k^2+\zeta x_k\cos (\Omega t)\Big ]+\frac{e^2}{4\pi \varepsilon (x_2-x_1)}, \nonumber \\ H_{\sigma }& = \sum _{k=1}^2\Big [(\alpha _D\sigma _k^x+\alpha _R\sigma _k^y) p_k+ g(\sigma _k^x\cos \theta +\sigma _k^y\sin \theta )\Big ]. \end{aligned}$$

Here $x_k$ and $p_k=-\text {i} \hbar \partial /\partial x_k$ are the position and momentum of *k*th impenetrable particle^29,38^ fulfilling $x_2>x_1$; $m_{eff}\sim 0.01 m_e$ and $\varepsilon \sim 10\varepsilon _0$ are the effective electron mass and dielectric constant^27^ with the electron mass $m_e$ and dielectric constant $\varepsilon _0$; *e* and $\omega$ denote the electron charge and the trap frequency, $\alpha _{R(D)}$ is the Rashba (Dresselhaus) SOC intensity, $\sigma _k^{x(y)}$ is the *x*(*y*) component of Pauli operator acting on the *k*th electron; the Zeeman term stands for $g=\frac{1}{2} g_e \mu _B B$ which contains the Landé factor $g_e$, the Bohr magneton $\mu _B$ and the controllable magnetic field strength *B*; $\theta$ represents the magnetic field orientation. The controllable amplitude $\zeta$ and frequency $\Omega$ of the ac electric field can be selected, respectively, in a wide region^55^. The harmonic oscillator level $\hbar \omega$ and quantum dot size are in orders of $(1\sim 10)$meV$\approx (1\sim 10)\hbar$THz and in $(1\sim 50)$nm respectively^11,12,17,27^.

In the basis^38^ $\{|\uparrow \uparrow \rangle , |\downarrow \downarrow \rangle , |\uparrow \downarrow \rangle , |\downarrow \uparrow \rangle \}$, the usual state vector of the system is $|\psi (t)\rangle =\sum _{i,j=1}^{2}|\psi _{\eta _i\eta _j}(t)\rangle |\eta _i\eta _j\rangle$ with $\eta _1=\uparrow , \eta _2=\downarrow$, and the space-dependent state vector reads

2 $$\begin{aligned} |\psi (x_1,x_2,t)\rangle =\langle x_1,x_2|\psi (t)\rangle =\sum _{i,j=1}^{2}\psi _{\eta _i\eta _j} (x_1,x_2,t)|\eta _i\eta _j\rangle , \end{aligned}$$

where the motional state function $\psi _{\eta _i\eta _j}(x_1,x_2,t)=\langle x_1,x_2|\psi _{\eta _i\eta _j}(t)\rangle$ is the coordinate representation of state vector $|\psi _{\eta _i\eta _j}(t)\rangle$. The square norm $|\psi _{\eta _i\eta _j} (x_1,x_2,t)|^2$ denotes the probability density of the particles being in spin states $|\eta _i\eta _j \rangle$, so the corresponding probability reads $P_{\eta _i\eta _j}(t)=\int \int |\psi _{\eta _i\eta _j} (x_1,x_2,t)|^2dx_1 dx_2.$ The internal spin state $|\eta _i\eta _j\rangle$ is identical to $|\eta _i\rangle _1|\eta _j\rangle _2$ with $|\eta _i\rangle _k$ being a single spin state of *k*th electron, including the spin-up state $$|\eta _1\rangle _k=|\uparrow \rangle _k=\left( \begin{array}{c} 1 \\ 0 \end{array}\right)$$ and spin-down state $$|\eta _2\rangle _k=|\downarrow \rangle _k=\left( \begin{array}{cc} 0 \\ 1 \end{array}\right)$$, respectively. The motional states may be expanded in terms of a set of orthonormal basic kets with time-dependent expansion coefficients^5^. The corresponding perturbed solution was also considered and some interesting results were found for a single-electron case^12^. However, hereafter we seek the exact orthonormalized complete solutions of Eq. (2). It is intractable but also worth, because of the more accurate results associated with the exact solutions.

Applying Eqs. (1) and (2) to the Schrödinger quation yields

3 $$\begin{aligned} \text {i}\hbar \frac{\partial |\psi (x_1,x_2,t)\rangle }{\partial t}= \text {i}\hbar \frac{\partial }{\partial t}\sum _{i,j=1}^{2}\psi _{\eta _i\eta _j} |\eta _i\eta _j\rangle =(H_0+H_{\sigma })\sum _{i,j=1}^{2}\psi _{\eta _i\eta _j} |\eta _i\eta _j\rangle . \end{aligned}$$

Making use of the well-known formulas $\sigma _k^x |\eta _i\rangle _k=|\eta _{i'}\rangle _k$ and $\sigma _k^y |\eta _j\rangle _k=(-1)^{j+1}\text {i} |\eta _{j'}\rangle _k$ for $i,i',j,j'=1,2$ and $i\ne i',j\ne j'$, we have the calculation

4 $$\begin{aligned} H_{\sigma }\sum _{i,j=1}^{2}\psi _{\eta _i\eta _j} |\eta _i\eta _j\rangle& = \sum _{i,j=1}^{2}\psi _{\eta _i\eta _j}\Big [(\alpha e^{(-1)^{i+1}\text {i} \phi }p_1+g e^{(-1)^{i+1}\text {i} \theta })|\eta _{i'}\eta _j\rangle \nonumber \\&\quad +(\alpha e^{(-1)^{j+1}\text {i} \phi }p_2+g e^{(-1)^{j+1}\text {i} \theta })|\eta _{i}\eta _{j'}\rangle \Big ]. \end{aligned}$$

Here we have adopted the expressions $\alpha e^{(-1)^{i+1}\text {i} \phi }=\alpha _D+(-1)^{i+1}\text {i} \alpha _R,\ e^{(-1)^{i+1}\text {i} \theta }=\cos \theta +(-1)^{i+1}\text {i}\sin \theta$ and^12^ $\alpha =\sqrt{\alpha _D^2+\alpha _R^2},\ \phi =\arctan \frac{\alpha _R}{\alpha _D}$. Combining Eqs. (3) and (4), we get

5 $$\begin{aligned} \text {i}\hbar \frac{\partial \psi _{\eta _i\eta _j}}{\partial t}=H_0\psi _{\eta _i\eta _j}+\Big [\alpha e^{(-1)^{i+1}\text {i} \phi }p_1+g e^{(-1)^{i+1}\text {i} \theta }\Big ]\psi _{\eta _{i'}\eta _j}+\Big [\alpha e^{(-1)^{j+1}\text {i} \phi }p_2+g e^{(-1)^{j+1}\text {i} \theta }\Big ]\psi _{\eta _i\eta _{j'}} \end{aligned}$$

for $i,i',j,j'=1,2$ and $i\ne i',j\ne j'$. This equation includes four coupled equations among four motional states, which is quite hard to analytically solve. However, by adjusting the orientation angle $\theta$ of magnetic field to match the SOC-dependent phase $\phi$, we can decouple them for constructing the exact solutions^52^. The match condition $\phi =\theta$ is experimentally feasible for the fixed SOC intensities $\alpha _R$ and $\alpha _D$, by selecting the proper orientation of magnetic field. Under such a condition, from Eq. (5) we arrive at the new coupled equations

$$\begin{aligned} \text {i}\hbar \frac{\partial }{\partial t}(\psi _{\eta _1\eta _1}+e^{2\text {i} \phi }\psi _{\eta _2\eta _2})& = H_0(\psi _{\eta _1\eta _1}+e^{2\text {i} \phi }\psi _{\eta _2\eta _2})+[\alpha (p_1+p_2)+2g]e^{\text {i} \phi }(\psi _{\eta _1\eta _2}+\psi _{\eta _2\eta _1}), \nonumber \\ \text {i}\hbar \frac{\partial }{\partial t}(\psi _{\eta _1\eta _2}+\psi _{\eta _2\eta _1})& = H_0(\psi _{\eta _1\eta _2} +\psi _{\eta _2\eta _1})+[\alpha (p_1+p_2)+2g]e^{-\text {i} \phi }(\psi _{\eta _1\eta _1}+e^{2\text {i} \phi }\psi _{\eta _2\eta _2}); \nonumber \\ \text {i}\hbar \frac{\partial }{\partial t}(\psi _{\eta _1\eta _1}-e^{2\text {i} \phi }\psi _{\eta _2\eta _2})& = H_0(\psi _{\eta _1\eta _1}-e^{2\text {i} \phi }\psi _{\eta _2\eta _2})+\alpha (p_2-p_1)e^{\text {i} \phi }(\psi _{\eta _1\eta _2}-\psi _{\eta _2\eta _1}), \nonumber \\ \text {i}\hbar \frac{\partial }{\partial t}(\psi _{\eta _1\eta _2}-\psi _{\eta _2\eta _1})& = H_0(\psi _{\eta _1\eta _2} -\psi _{\eta _2\eta _1})+\alpha (p_2-p_1)e^{-\text {i} \phi }(\psi _{\eta _1\eta _1}-e^{2\text {i} \phi }\psi _{\eta _2\eta _2}) \end{aligned}$$

among the four combined motional states. Given these equations, we can multiply the first and third equations by $e^{-\text {i} \phi /2}$ and multiply the second and fourth equations by $e^{\text {i} \phi /2}$, then combine the first with the second, and the third with the fourth, respectively, to obtain the decoupled equations

6 $$\begin{aligned} \text {i}\hbar \frac{\partial \Psi _k}{\partial t}& = H_0\Psi _k+(-1)^{k+1}[\alpha (p_1+p_2)+2g]\Psi _k, \nonumber \\ \Psi _k(x_1,x_2,t)& = (\psi _{\eta _1\eta _1}+e^{2\text {i} \phi }\psi _{\eta _2\eta _2})e^{-\text {i} \phi /2}+(-1)^{k+1}(\psi _{\eta _1\eta _2}+\psi _{\eta _2\eta _1})e^{\text {i} \phi /2}; \nonumber \\ \text {i}\hbar \frac{\partial \Phi _k}{\partial t}& = H_0\Phi _k+(-1)^{k+1}\alpha (p_2-p_1)\Phi _k, \nonumber \\ \Phi _k(x_1,x_2,t)& = (\psi _{\eta _1\eta _1}-e^{2\text {i} \phi }\psi _{\eta _2\eta _2})e^{-\text {i} \phi /2}+(-1)^{k+1}(\psi _{\eta _1\eta _2}-\psi _{\eta _2\eta _1})e^{\text {i} \phi /2},\nonumber \\ \theta =\phi& = \arctan \frac{\alpha _R}{\alpha _D}=\phi _0+l\pi \ \ \ \text{ for } \ \ \ k=1,2;\ l=0,1,2,\ldots \end{aligned}$$

with $\phi _0\in [0,\pi /2]$, which contains the case $\phi =\theta =0$ of Rashba SOC vanishing.

Now we seek the separable solutions of Eq. (6) in the forms

7 $$\begin{aligned} \Psi _k(x_1,x_2,t)=\Psi _k^c(x_c,t)\Psi _k^r(x_r,t),\ \ \Phi _k(x_1,x_2,t)=\Phi _k^c(x_c,t)\Phi _k^r(x_r,t) \end{aligned}$$

with the center-of-mass and relative coordinates $x_c=\frac{1}{2} (x_2+x_1),\ p_c=-\text {i} \hbar \partial /\partial x_c =(p_2+p_1)$ and $x_r=x_2-x_1,\ p_r=-\text {i} \hbar \partial /\partial x_r=\frac{1}{2} (p_2-p_1)$. In the new coordinate system, $H_0$ of Eq. (6) becomes

8 $$\begin{aligned} H_0(x_c,x_r,t)=\sum _{\beta =c}^r\Big [-\frac{p_{\beta }^2}{2m_{\beta }}+\frac{1}{2} m_{\beta }\omega ^2 x_{\beta }^2\Big ]+\zeta x_c\cos (\Omega t)+\frac{e^2}{4\pi \varepsilon x_r}, \end{aligned}$$

where $m_c=2m_{eff}$ and $m_r=m_{eff}/2$. Application of Eqs. (7) and (8) to Eq. (6) produces

9 $$\begin{aligned} \text {i}\frac{\partial \Psi _k^c}{\partial t}& = \Big [-\frac{p_c^2}{2}+\frac{1}{2} x_c^2+\zeta x_c\cos (\Omega t)+(-1)^{k+1}(\alpha p_c+2g)\Big ]\Psi _k^c, \nonumber \\ \text {i}\frac{\partial \Psi _k^r}{\partial t}& = \Big [-\frac{p_r^2}{2}+\frac{1}{2} x_r^2+\frac{\sigma }{2 x_r}\Big ]\Psi _k^r; \nonumber \\ \text {i}\frac{\partial \Phi _k^c}{\partial t}& = \Big [-\frac{p_c^2}{2}+\frac{1}{2} x_c^2+\zeta x_c\cos (\Omega t)\Big ]\Phi _k^c, \nonumber \\ \text {i}\frac{\partial \Phi _k^r}{\partial t}& = \Big [-\frac{p_r^2}{2}+\frac{1}{2} x_r^2+\frac{\sigma }{2 x_r}+(-1)^{k+1}2\alpha p_r \Big ]\Phi _k^r. \end{aligned}$$

Here $x_c$ and $x_r$ have been normalized in units of $a_c=\sqrt{\hbar /(m_c\omega )}$ and $a_r=\sqrt{\hbar /(m_r\omega )}=2a_c$, the frequency $\Omega$, time *t* and energy are in units of $\omega$, $\omega ^{-1}$ and $\hbar \omega$, respectively. The parameters $\zeta ,\ \alpha$ and *g* have also been normalized correspondingly. The parameter $\frac{e^2}{\varepsilon }$ is associated with the dimensionless one $\sigma =\frac{e^2}{4\pi \varepsilon \hbar \omega a_r}=\frac{e^2}{4\pi \varepsilon }\sqrt{\frac{2m_{eff}}{\hbar ^3\omega }}$ which expresses the importance of the Coulomb potential compared to the harmonic level and is confined by the exact solution of the relative motion^44–47^. The smaller $\sigma$ value corresponds to a greater trapping frequency $\omega$. The exact solutions of the second and the third of Eq. (9) are well-known for us. The first (the fourth) of Eq. (9) can be changed to the similar form with the third (the second) of Eq. (9), by the function transformations

10 $$\begin{aligned} \Psi _k^c(x_c,t)& = C_ke^{\text {i} [(-1)^k(\alpha x_c+ 2gt) -\alpha ^2t/2)]}f_{n_k}(x_c,t),\ \ \Psi _k^r(x_r,t)=F_{n'_k}(x_r,t);\nonumber \\ \Phi _k^c(x_c,t)& = C_{k+2}f_{n_{k+2}}(x_c,t),\ \ \Phi _k^r(x_r,t)=e^{\text {i} [(-1)^k 2\alpha x_r-2\alpha ^2 t]}F_{n'_{k+2}}(x_r,t). \end{aligned}$$

Substituting Eq. (10) into Eq. (9), we arrive at the above-mentioned similar forms

11 $$\begin{aligned} \text {i}\frac{\partial f_{n_{k'}}(x_c,t)}{\partial t}& = \Big [-\frac{p_c^2}{2}+\frac{1}{2} x_c^2+\zeta x_c\cos (\Omega t)\Big ]f_{n_{k'}}(x_c,t), \nonumber \\ \text {i}\frac{\partial F_{n'_{k'}}(x_r,t)}{\partial t}& = \Big [-\frac{p_r^2}{2}+\frac{1}{2} x_r^2+\frac{\sigma }{2 x_r}\Big ]F_{n'_{k'}}(x_r,t) \end{aligned}$$

with $k'=k, k+2=1,2,3,4$. For different $k'$, functions $f_{n_{k'}}$ may be the same or different solutions of the first of Eq. (11), and $F_{n'_{k'}}$ may be the same or different solutions of the second of Eq. (11). To simplify, we will drop the sign “$'$” to write $k'$ as *k* in the following.

The first of Eq. (11) is a driven harmonic oscillator equation with the exact complete solution describing the orthonormal generalized coherent states^49,50^

12 $$\begin{aligned} f_{n_k}& = R_{n_k}(x_c,t)e^{\text {i}\Theta _{n_k}(x_c,t)}, \nonumber \\ \Theta _{n_k}& = -\Big (\frac{1}{2}+n_k \Big )\chi (t)+b_{k 2}x_c+\frac{{\dot{\rho }}}{2\rho }x_c^2+\gamma _k (t), \nonumber \\ R_{n_k}& = \Big (\frac{\sqrt{c_0}}{\sqrt{\pi }2^{n_k}n_{k}!\rho } \Big )^{\frac{1}{2}}H_{n_k}(\xi _k)e^{-\frac{1}{2} \xi ^2_k}, \nonumber \\ \xi _k& = \frac{\sqrt{c_0}}{\rho (t)}x_c-\frac{b_{k 1}(t)\rho (t)}{\sqrt{c_0}},\ \ \ \end{aligned}$$

for $k=1,2,3,4;\ n_k=0,1,2,\ldots$, where $R_{n_k}(x_c,t)$ and $\Theta _{n_k}(x_c,t)$ are the real functions and $H_{n_k}(\xi _k)$ the Hermite polynomial of the space-time combined variable $\xi _{k}(x_c,t)$. In Eq. (12), the real functions $\rho (t), \chi (t)$, $\gamma _{k}(t), b_{k 1}(t)$ and $b_{k 2}(t)$ implied in $\gamma _k (t)$ have the known forms^49,50^

$$\begin{aligned} \rho (t)& = \sqrt{\varphi _1^2+\varphi _2^2}, \ \ \chi (t)=\arctan \Big (\frac{\varphi _2}{\varphi _1}\Big ),\ \ \varphi _{1,2}(t)=A_{1,2} \cos (t+B_{1,2}), \\ \gamma _{k}(t)& = \frac{1}{2}\int _0^t[b_{k 1}^2(\tau )-b_{k 2}^2(\tau )]d\tau +\gamma _{k}(0), \\ b_{k 1}(t)& = \frac{\zeta }{\rho ^2(t)}\Big [\varphi _1 (t)\int _0^t \varphi _2(\tau )\cos (\Omega \tau )d \tau -\varphi _2(t) \int _0^t \varphi _1(\tau )\cos (\Omega \tau )d \tau \Big ]\\&\quad +b_{k 1}(0)\varphi _1(t)+b_{k 2}(0)\varphi _2(t), \\ b_{k 2}(t)& = \frac{\zeta }{\rho ^2(t)}\Big [-\varphi _1(t) \int _0^t \varphi _1(\tau )\cos (\Omega \tau )d \tau -\varphi _2(t) \int _0^t \varphi _2(\tau )\cos (\Omega \tau )d \tau \Big ]\\& \quad +b_{k 2}(0)\varphi _1(t)+b_{k 1}(0)\varphi _2(t). \end{aligned}$$

Here $c_0={{\dot{\varphi }}}_2 \varphi _1-{{\dot{\varphi }}}_1 \varphi _2$ is a constant adjusted by the constants $A_{1,2}$ and $B_{1,2}$. Given the driving parameters $\zeta ,\Omega$ and the quantum numbers $n_k$, the initial-state-dependent constant sets $\{S_k\}=\{\gamma _k(0), b_{k 1}(0), b_{k 2}(0),A_{1,2}, B_{1,2}\}$ are determined by the forms of the initial states^51^. Then the solutions $f_{n_k}(S_k,x_c,t)$ are definite for $n_k=0,1,2,\ldots$, respectively.

The second of Eq. (11) is a harmonic-Coulomb competition system whose exact stationary-state solutions are also well-known for us^47^, that is

13 $$\begin{aligned} F_{n'_k}(x_r,t)& = A_{n'_k} e^{-\text {i} E^r_{n'_{k}} t}F_{n'_k} (x_r)=A_{n'_k}e^{-\text {i} E^r_{n'_k} t-x_r^2/2} \sum _{j=0}^{n'_k}D_jx_r^{j+1}, \nonumber \\ E^r_{n'_k}(\omega )& = \Big (\frac{3}{2} +n'_k\Big )\hbar \omega ,\ \ \omega =\omega _{n'_k}=\Big (\frac{e^2}{4\pi \varepsilon \sigma _{n'_k}}\Big )^2\frac{2 m_{eff}}{\hbar ^3} \end{aligned}$$

for $k=1,2,3,4$ and $n'_k=1,2,\ldots$, where $A_{n'_k}$ is a normalization constant. Note that $n'_k$ is a pseudo quantum-number and is fixed to a single integer for an experimentally given trapping frequency $\omega _{n'_k}$. The dimensionless constants $\sigma _{n'_k}$ and $D_j$ are determined by the algebraic equations^47^

$$\begin{aligned} 2D_{n'_k-1}-\sigma _{n'_k}D_{n'_k}=0,\ (n'_k-j)(n'_k-j+1)D_{n'_k-j}-\sigma _{n'_k}D_{n'_k-j-1}+2(j+2)D_{n'_k-j-2}=0 \end{aligned}$$

for $n'_k=1,2,\ldots;\ j=0,1,2,\ldots,n'_k-1$ and $D_0=1, D_{j<1}=0$. In the simplest case $n'_k=1$, these equations give^47^ $D_1=D_0=1$ and the minimal constant $\sigma _1=2$ associated with the maximal trapping frequency $\omega _1\sim 10^{12}$Hz for the smaller effective mass^27^ $m_{eff}\sim 0.01 m_e$ and the larger dielectric constant $\varepsilon \sim 10\varepsilon _0$. Such trapping frequency and the corresponding harmonic oscillator length $a_c$ in order of 10nm are experimentally realizable^11,27^.

Given Eqs. (6), (7) and (10), we derive the exact motional states

14 $$\begin{aligned} \psi _{\eta _j\eta _j}& = \frac{1}{4} e^{\text {i}(\frac{\phi }{2} -\delta _{2j}2\phi )}[(\Psi _1+\Psi _2)+(-1)^{j+1}(\Phi _1+\Phi _2)]\nonumber \\& = \frac{1}{4} e^{\text {i}(\frac{\phi }{2}-\delta _{2j}2\phi -\frac{\alpha ^2}{2}t)}\Big [(C_1e^{-\text {i} (\alpha x_c+2gt)} f_{n_1}F_{n'_1}+C_2e^{\text {i} (\alpha x_c+2gt)}f_{n_2}F_{n'_2})\nonumber \\&\quad +(-1)^{j+1} e^{-\text {i}\frac{3\alpha ^2}{2}t} (C_3e^{-\text {i} 2\alpha x_r}f_{n_3}F_{n'_3}+C_4e^{\text {i} 2 \alpha x_r}f_{n_4}F_{n'_4})\Big ], \nonumber \\ \psi _{\eta _j\eta _{j'}}& = \frac{1}{4} e^{-\text {i}\frac{\phi }{2}} {[}(\Psi _1-\Psi _2)+(-1)^{j+1}(\Phi _1-\Phi _2)]\nonumber \\& = \frac{1}{4} e^{-\text {i}(\frac{\phi }{2}+\frac{\alpha ^2}{2}t)} \Big [(C_1e^{-\text {i} (\alpha x_c+2gt)}f_{n_1}F_{n'_1}-C_2e^{\text {i} (\alpha x_c+2gt)}f_{n_2}F_{n'_2})\nonumber \\&\quad +(-1)^{j+1} e^{-\text {i}\frac{3\alpha ^2}{2}t}(C_3e^{-\text {i} 2\alpha x_r}f_{n_3}F_{n'_3}-C_4e^{\text {i} 2\alpha x_r}f_{n_4}F_{n'_4})\Big ] \end{aligned}$$

for $j=1,2,\ j\ne j'$ and $\delta _{21}=0,\ \delta _{22}=1$, where $f_{n_k}=f_{n_k}(x_c,t)$ and $F_{n'_k}=F_{n'_k}(x_r,t)$ are given in Eqs. (12) and (13). The solutions of Eq. (14) stand for a set of exact complete solutions of Eq. (5) with the initial-state-dependent constants $\{C_k\}$ and $\{S_k\}$ implied in $f_{n_k}$. They contain the four motional states $\psi _{\eta _1\eta _1}=\psi _{\uparrow \uparrow }(x_c,x_r,t),\ \psi _{\eta _2\eta _2}=\psi _{\downarrow \downarrow }(x_c,x_r,t),\ \psi _{\eta _1\eta _2}=\psi _{\uparrow \downarrow }(x_c,x_r,t)$ and $\psi _{\eta _2\eta _1}=\psi _{\downarrow \uparrow }(x_c,x_r,t)$. Any one of them can be regarded as a *coherent superposition of the generalized coherent states* $f_{n_k}(x_c,t)$ with $C_kF_{n'_k}(x_r,t)$ and the corresponding exponent functions being the superposition coefficients. For the orthonormal generalized coherent states $f_{n_k}(x_c,t)$ and the stationary states $F_{n'_k}(x_r,t)$, we can prove that the corresponding state vector (2) satisfies the orthonormalization condition. In order to simplify the calculations, hereafter we consider only the simple case $f_{n_k}=f_n (x_c,t),\ F_{n'_k}=F_{n'}(x_r,t)=Ae^{\text {i} E^r_{n'} t}F_{n'}(x_r)$ for $k=1,2,3,4$. Thus the state vector (2) and the motional states (14) can be labeled by the quantum number *n* and pseudo quantum-number $n'$, $|\psi (x_1,x_2,t)\rangle =|\psi _{nn'}(x_c,x_r,t)\rangle$ and $\psi _{\eta _i\eta _j}=\psi _{\eta _i\eta _j,nn'}(x_c,x_r,t)$ with the constant set $\{S_k\}=\{S\}=\{\gamma (0), b_{1}(0), b_{2}(0),A_{1,2}, B_{1,2}\}$ of Eq. (12), which obey the *orthonormalization condition*

15 $$\begin{aligned}&\langle \psi _{nn'}(x_c,x_r,t)|\psi _{mn'}(x_c,x_r,t)\rangle =\sum _{i,j=1}^{2}\int _{-\infty }^{\infty }dx_c\int _0^{\infty } dx_r \psi ^*_{\eta _i\eta _j,nn'}\psi _{\eta _i\eta _j,mn'}\nonumber \\&=\frac{4}{16}\sum _{k=1}^{4}|C_k|^2e^{\text {i} (n-m)t} \int _{-\infty }^{\infty } |f_n (x_c,t)f_m (x_c,t)| dx_c \int _0^{\infty } |F_{n'}(x_r,t)|^2 dx_r\nonumber \\&= \frac{A^2}{4}\sum _{k=1}^{4}|C_k|^2e^{\text {i} (n-m)t}\delta _{nm} \int _0^{\infty }|F_{n'}(x_r)|^2 dx_r=e^{\text {i} (n-m)t}\delta _{nm}, \nonumber \\&A^2=4 /\Big [\sum _{k=1}^{4}|C_k|^2 \int _0^{\infty }|F_{n'}(x_r)|^2 dx_r\Big ]. \end{aligned}$$

The careful calculation gives the expected energy of state $|\psi _{nn'}(x_c,x_r,t)\rangle$ as^49,50,52^

16 $$\begin{aligned} E_{nn'}& = \langle \psi _{nn'}(x_c,x_r,t)|\text {i} \frac{\partial }{\partial t}\psi _{nn'}(x_c,x_r,t)\rangle \nonumber \\& = \text {i} \sum _{i,j=1}^{2}\int _{-\infty }^{\infty }\int _0^{\infty } \psi ^*_{\eta _i\eta _j,nn'}\frac{\partial \psi _{\eta _i\eta _j,nn'}}{\partial t}dx_c dx_r \nonumber \\& = \frac{\alpha ^2}{2}+\text {i} 4 \frac{A^2}{4^2}\int _{-\infty }^{\infty } \int _0^{\infty }\Big [\sum _{k=1}^2(C_ke^{(-1)^k\text {i} (\alpha x_c+2gt)} f_{n}F_{n'})^*\frac{\partial }{\partial t}\sum _{k=1}^2(C_ke^{(-1)^k \text {i} (\alpha x_c+2gt)}f_{n}F_{n'}) \nonumber \\& \quad +\sum _{k=3}^4(C_ke^{\text {i}[(-1)^k2\alpha x_r-\frac{3\alpha ^2}{2}t]}f_{n}F_{n'})^*\frac{\partial }{\partial t}(C_ke^{\text {i} [(-1)^k2 \alpha x_r-\frac{3\alpha ^2}{2}t]}f_{n}F_{n'})\Big ]dx_c dx_r \nonumber \\& = \frac{\alpha ^2}{2}+E_{n'}^r+\frac{A^2}{4}\Big [(|C_1|^2-|C_2|^2)2g +(|C_3|^2+|C_4|^2)\frac{3\alpha ^2}{2}\Big ]\int _0^{\infty }|F_{n'}(x_r) |^2 dx_r \nonumber \\& \quad +\text {i} \frac{A^2}{4} \sum _{k=1}^4 |C_k|^2\int _{-\infty }^{\infty } f_n^*(x_c,t)\frac{\partial f_n (x_c,t)}{\partial t}dx_c \int _0^{\infty }|F_{n'}(x_r)|^2 dx_r \nonumber \\& = \frac{\alpha ^2}{2}+E_{n'}^r+\Big (\sum _{k=1}^4 |C_k|^2 \Big )^{-1} \Big [(|C_1|^2-|C_2|^2)2g+(|C_3|^2+|C_4|^2)\frac{3\alpha ^2}{2}\Big ] +E_{cn}(t), \nonumber \\ E_{cn}(t)& = \frac{1}{2} +n+\frac{1}{2} [x_{cn}^2(t)+ p_{cn}^2(t)]+\zeta x_{cn}(t) \cos (\Omega t), \end{aligned}$$

where $x_{cn}(t)=\int _{-\infty }^{\infty } x_c |f_n(x_c,t)|^2 dx_c$ and $p_{cn}(t)={\dot{x}}_{cn}$ denote the expectation values of coordinate $x_c$ and momentum $p_c$. Clearly, the energy $E_{cn}(t)$ consists of a quantum part $\frac{1}{2} +n$ and a continuously time-varying one. For an undriven coherent state with $\zeta =0$, $E_{cn}$ is equal to a constant, although $x_{cn}(t)$ and $p_{cn}(t)$ are time-dependent.

It is worth noting that Eq. (12) is valid for any experimentally realizable trapping frequency, but the validation of Eq. (13) is associated with only the fixed trapping frequency $\omega _{n'_k}=\omega _{n'}$. Therefore, for a given trapping frequency the pseudo quantum-number $n'_k=n'$ and the relative energy $E^r_{n'_k}(\omega _{n'_k})=E^r_{n'}(\omega _{n'})$ are fixed, and for two determined initial-state-dependent constant sets $\{S_k\}$ and $\{C_k\}$ the quantum levels of $|\psi _{nn'}\rangle$ are distinguished only by the quantum number $n=0,1,\ldots$. The different initial constant sets can correspond to the different ground states $|\psi _{0n'}\rangle$ with the lowest vibrational quantum number $n=0$ and the corresponding instantaneous energies $E_{0n'}$. By the instantaneous degenerate ground states^40^ we mean that they correspond to the different initial constant sets $\{S_k\},\ \{C_k\}$ and the same instantaneous energy $E_{c0}(t)$ given by Eq. (16). Applying Eqs. (14) and (15), we can transparently perform coherent manipulations, by preparing appropriate initial states and adjusting the control parameters. We will take the non-degenerate ground state with $n=0$ and the trapping frequency $\omega _{n'}=\omega _1$ as an example as follows.

### Transparently coherent manipulation to probability densities occupying spin states

In the ground state case with $n_k=n=0,\ n'_k=n'=1$ and $f_{n_k}F_{n'_k}=f_0F_1$, the probability densities are described by the square norms $|\psi _{\eta _i\eta _j,01}(x_c,x_r,t)|^2$ of the motional states given in Eq. (14),

17 $$\begin{aligned} |\psi _{\eta _j\eta _j,01}|^2& = \frac{1}{16} \Big |C_1e^{-\text {i} (\alpha x_c+2gt)}+C_2e^{\text {i} (\alpha x_c+2gt)}+(-1)^{j+1} e^{-\text {i}\frac{3\alpha ^2}{2}t} (C_3e^{-\text {i} 2\alpha x_r}+C_4e^{\text {i} 2\alpha x_r})\Big |^2 \nonumber \\&\times |f_0(x_r,t)F_1(x_r)|^2, \nonumber \\ |\psi _{\eta _j\eta _{j'},01}|^2& = \frac{1}{16} \Big |C_1e^{-\text {i} (\alpha x_c+2gt)}-C_2e^{\text {i} (\alpha x_c+2gt)}+(-1)^{j+1} e^{-\text {i}\frac{3\alpha ^2}{2}t} (C_3e^{-\text {i} 2\alpha x_r}-C_4e^{\text {i} 2\alpha x_r})\Big |^2\nonumber \\&\times |f_0(x_r,t)F_1(x_r)|^2 \end{aligned}$$

for $j=1,2,\ j\ne j'$. We select the constant set^49,50^ $\{S\}=\{\gamma (0)= b_2(0)=0, b_1(0)=x_0/\sqrt{c_0},\ A_1=A_2=\sqrt{c_0}, B_1=0,B_2=-\pi /2\}$, then Eq. (12) and its auxiliary equations give the functions $f_0(x_c,t)$ and $\Theta _0(x_c,t)$ as the following

18 $$\begin{aligned} \varphi _1(t)& = \sqrt{c_0}\cos t,\ \ \varphi _2(t)=\sqrt{c_0}\sin t,\ \ \rho =\sqrt{c_0},\ \ \chi (t)= t,\ \ \xi _k=x_c-b_1(t), \nonumber \\ b_1(t)& = \zeta \Big [\cos t \int _0^t \sin \tau \cos (\Omega \tau )d \tau -\sin t\int _0^t \cos \tau \cos (\Omega \tau )d \tau \Big ]+x_0\cos t, \nonumber \\ b_2(t)& = \zeta \Big [-\cos t \int _0^t \cos \tau \cos (\Omega \tau )d \tau -\sin t \int _0^t \sin \tau \cos (\Omega \tau )d \tau \Big ]+x_0\sin t, \nonumber \\ f_{n_k}(x_c,t)& = f_0(x_c,t)=\pi ^{-1/4}e^{-(x_c-b_1)^2/2}e^{\text {i} \Theta _0(x_c,t)}\ \ \ \text {for} \ \ \ k=1,2,3,4, \nonumber \\ \Theta _0(x_c,t)& = -\frac{1}{2}t+b_2x_c+\frac{1}{2}\int _0^t[b_1^2(\tau ) -b_2^2(\tau )]d\tau . \end{aligned}$$

In addition, for $n'_k=n'=1$ and $D_0=D_1=1$, Eqs. (13) and (15) give the function

19 $$\begin{aligned} F_{n'}(x_r,t)& = F_1(x_r,t)=A e^{-\text {i} E^r_1 t}F_1(x_r) =Ae^{-\text {i} E^r_1 t-x_r^2/2}(x_r+x_r^2), \nonumber \\ A^2& = 4 /\Big [\sum _{k=1}^{4}|C_k|^2 \int _0^{\infty }|F_1(x_r)|^2 dx_r\Big ]=1.8977/\sum _{k=1}^{4}|C_k|^2. \end{aligned}$$

From Eqs. (15), (18) and (19) we derive the expected spatial coordinates^49^

20 $$\begin{aligned} x_{cn}(t)& = x_{c0}(t)=\int _{-\infty }^{\infty } x_c |f_0(x_c,t)|^2 dx_c =b_1(t), \nonumber \\ x_{r1}& = \frac{A^2}{4} \sum _{k=1}^4 |C_k|^2\int _0^{\infty }x_r |F_{1}(x_r)|^2 dx_r \nonumber \\& = \frac{\int _0^{\infty }x_r|F_{1}(x_r)|^2 dx_r}{\int _0^{\infty }|F_{1}(x_r)|^2 dx_r}=1.3423, \end{aligned}$$

which means that the center-of-mass of the two electrons undergos a motion just like a classically driven harmonic oscillator^49^, and the distance $x_{r1}$ between electrons is a constant. Making use of the relations between $(x_1, x_2)$ and $(x_c, x_r)$, from Eq. (20) we get the electronic positions $x_1=b_1(t)-\frac{x_{r1}}{2}$ and $x_2=b_1(t)+\frac{x_{r1}}{2}$. In Eq. (17), we observe that intensities of the SOC and magnetic field appear in the phases of $\psi _{\eta _i\eta _j,01}$, which can be used to tune the coherence terms of the probability densities and to perform the coherent control of the system.

#### Manipulating spatial distributions of the probability densities via SOC

**Figure 1:**
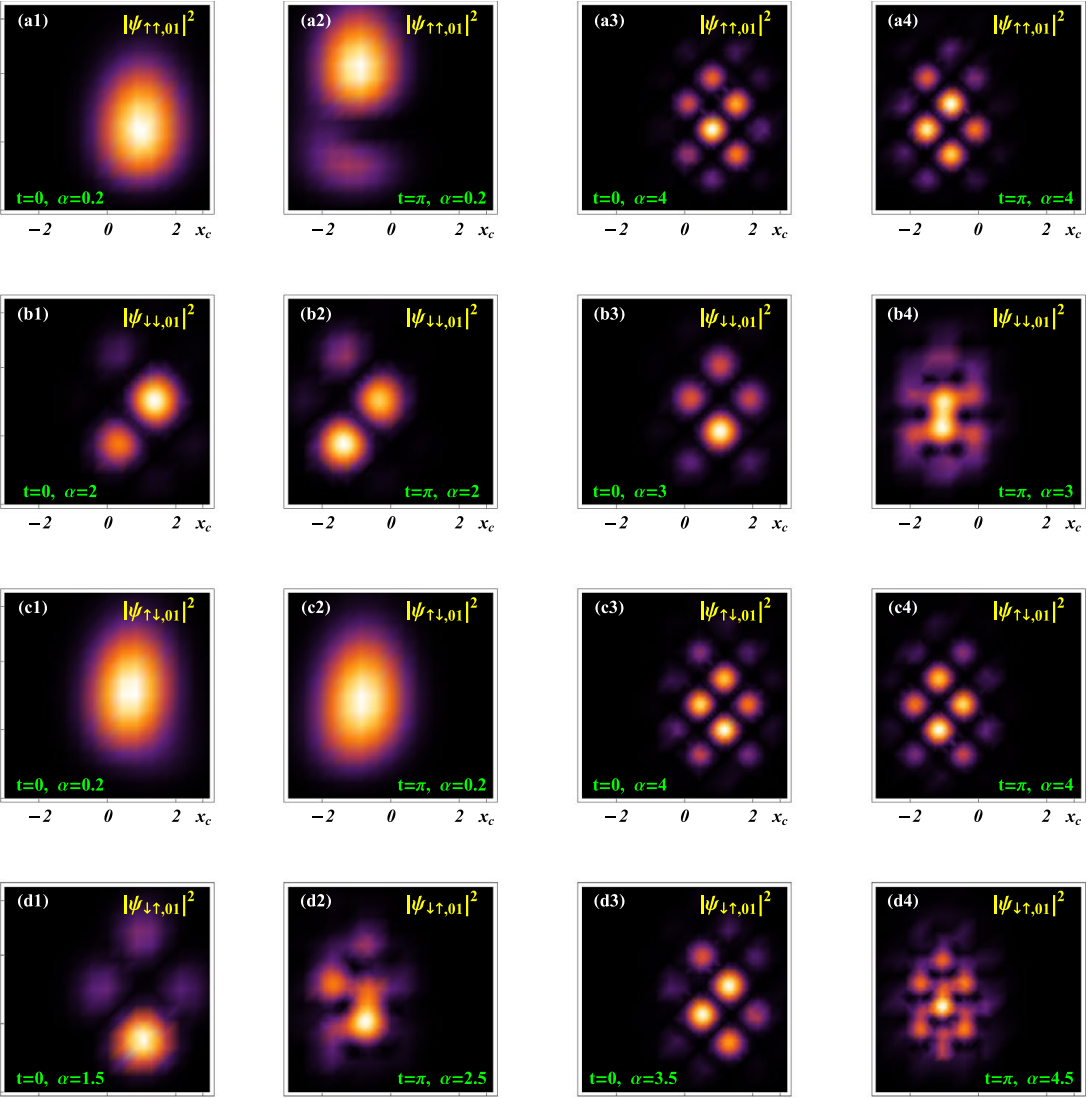
Spatial distributions of the probability density components $|\psi _{\eta _i\eta _j,01}|^2$ at $t=0,\ \pi$ for the initial constants $\{C_k\} = (C_ 1, C_ 2, C_ 3, C_ 4) = (0.45, 0.55, 0.35, 0.75)$ and $x_0 = 1$, and the system parameters $\zeta = 0, \Omega = 0, g = 0.5$. (**a**) $|\psi _{\uparrow \uparrow ,01}|^2$ with $\alpha = 0.2$ in (a1) and (a2), and with $\alpha = 4$ in (a3) and (a4); (**b**) $|\psi _{\downarrow \downarrow ,01}|^2$ with $\alpha = 2$ in (b1) and (b2), and with $\alpha =3$ in (b3) and (b4); (**c**) $|\psi _{\uparrow \downarrow ,01}|^2$ with $\alpha = 0.2$ in (c1) and (c2), and with $\alpha = 4$ in (c3) and (c4); (**d**) $|\psi _{\downarrow \uparrow ,01}|^2$ with $\alpha =1.5,2.5,3.5,4.5$ in (d1), (d2), (d3) and (d4), respectively. By this figure we show that shapes of the density wavepackets can change in the time evolution, and numbers and locations of the wave peaks depend mainly on SOC intensity. In Figs. 1 and 2, the lighter areas correspond to the higher densities and the lightest points of different regions indicate the density peaks of different heights, while a deeper colour area denotes some lower densities and the darkest area means the zero density and wavepacket spread. Hereafter, the probability density has been normalized in units of $1/(a_c a_r)$ and all the variables and parameters appearing in the figures are dimensionless.

Based on Eqs. (17–20), we employ the “Density Plot” of the Mathematica procedure to illustrate the coherent manipulation to the spatial distributions of probability density components, as shown in Fig. 1 for a set of fixed initial constants and the undriven case. Hereafter, the parameters *g* and $\alpha$ are taken in the intervals^11^ $g\in [0, 1)$ and^12^ $\alpha \in [0,5)$, respectively. It is shown that any density component describes some wavepackets with the different numbers and locations of the wave peaks. The wavepackets are discrete in the usual cases except for those of Figs. (b4), (d2) and (d4) with packet overlaps. Their center positions move from $(x_r=x_{r1} = 1.3423, x_c > 0)$ to $(x _{r1}, x_c < 0)$ with the increase of time from $t =0$ to $t = \pi$. Their peak numbers change between 1 and 10 for the given $\alpha$ values. We take $\alpha =0.2$ and 4 in (a) and (c); $\alpha = 2, 3$ and $\alpha =1.5, 2.5, 3.5, 4.5$ in (b) and (d), respectively, to shown that *the numbers of wave peaks depend mainly on SOC intensity*, the larger $\alpha$ value corresponds to more wave peaks. For the same $\alpha$ value, the wavepackets of different components, e.g. $|\psi _ {\uparrow \uparrow ,01} | ^2$ in (a) and $|\psi _{\uparrow \downarrow ,01}|^2$ in (c), exist distinguishable differences of the spatial distribution at the same time and on the same spatial region. The number and location of peaks and the shapes of wavepackets can change in the time evolution. The similar result is found for the components $|\psi _ {\downarrow \downarrow , 01}| ^2$ and $|\psi _ {\downarrow \uparrow ,01}|^2$, which is not exhibited here. The accurate manipulation to the wavepackets may be useful for performing a two-qubit quantum gate, referring to the case of a two-ion system^37^.

#### Controlling spatiotemporal evolutions of the probability densities via periodic driving

**Figure 2:**
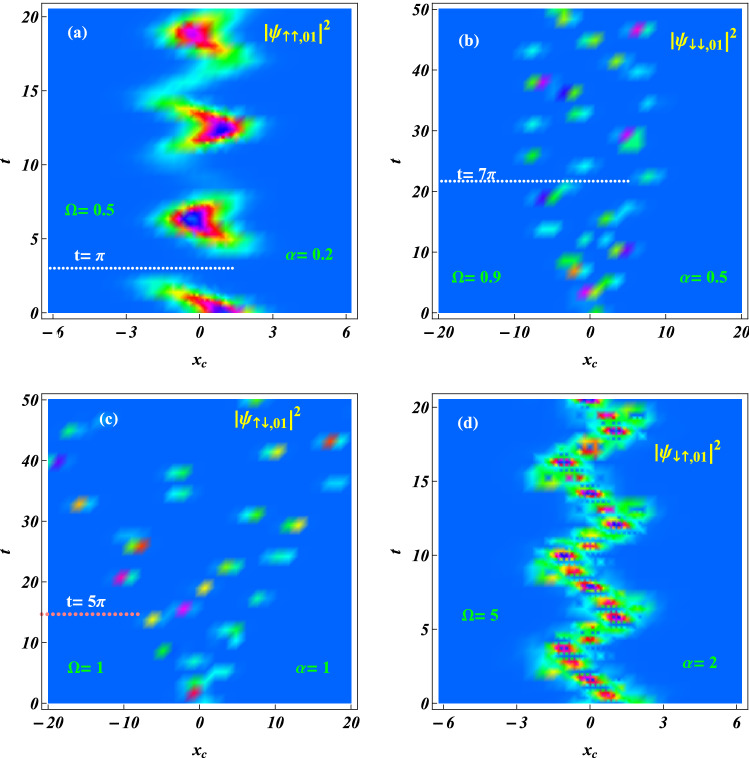
Spatiotemporal evolutions of the probability density components (**a**) $|\psi _ {\uparrow \uparrow , 01} | ^2$, (**b**) $|\psi _ {\downarrow \downarrow ,01}|^2$, (**c**) $|\psi _ {\uparrow \downarrow ,01}|^2$ and (**d**) $| \psi _ {\downarrow \uparrow ,01}|^2$ with the same constant set $\{C_k\}$ as that of Fig. 1. The parameters are selected as $\zeta = 1, x_r = x _{r1} = 1.3423, x_ 0 = 1, g= 0.5$ and (**a**) $\Omega = 0.5,\alpha = 0.2$; (**b**) $\Omega = 0.9, \alpha =0.5$; (**c**) $\Omega = 1, \alpha = 1$; and (**d**) $\Omega = 5, \alpha = 2$. It is illustrated that the spatial size of the density distribution depends on the frequency resonance effect and increasing the SOC intensity value can avoid appearance of the zero density component at any time.

The previous investigation demonstrated that for a charged two-particle system adopting periodic driving including the state-dependent forces to manipulate the probability density wavepackets could be used to implement a two-qubit phase gate^38,40^, where the Coulomb interaction is negligible. Here our exact solution is of the Coulomb-harmonic system (1) with a set of specific trapping frequencies. Notice that the linear combinations $\xi =x_c - b_1 (t)$ in the exponent function of Eq. (18) and the periodic driving implied in function $b_1 (t)$, we can employ the periodic driving to manipulate the spatiotemporal evolutions of the probability density components. In order to conveniently discuss the spatiotemporal evolutions and noticing the time-independence of the expected relative coordinate in Eq. (20), we consider a fixed value $x_r = x_{r1} =1.3423$ to plot the density components as the functions of $x_c$ and *t* in Fig. 2 . From Fig. 2(a) we observe that for the smaller parameter values $\Omega =0.5, \alpha = 0.2$, the component $|\psi _ {\uparrow \uparrow ,01}|^2$ oscillates in the small spatial interval $x_c \in (-3, 3)$ and moves in time with period being about $4\pi \gg \Omega$. At any time and for the fixed $x_r$ and arbitrary $x_c$, only one dispersed wavepacket exists, except for some moments at which the density component vanishes, as indicated by the dotted line at $t=\pi$. The zero density means zero probability of the electrons occupying spin state $|\uparrow \uparrow \rangle$, and is similar to the case of Fig. 1(a2). In Fig. 2(b) we can see that with increasing parameter values to $\Omega =0.9$ and $\alpha = 0.5$, the density component $|\psi _{\downarrow \downarrow ,01}|^2$ increases its time period to about $2\times 50$ and spatial region to $x_c\in (-10, 10)$. The time points of zero density still exist, as indicated by the line at $t= 7\pi$. Further increasing the driving frequency to $\Omega =1$ and the SOC intensity to $\alpha = 1$, in Fig. 2(c), we illustrate the effect of resonance on the spatiotemporal evolutions. In this case, we find that the distribution width of the density $|\psi _{\uparrow \downarrow , 01} |^2$ linearly increases without limitation. The linear resonance diffusion is related to the aperiodic expected coordinate^50^ $x_{c0} =b_1 (t)$ with one term being proportional to time *t*, as the second integral of $b_1 (t)$ in Eq. (18) with $\Omega =1$. At about $t =5\pi$ the distribution width reaches the size $|x_c| =10$ of the quantum dot. This means that the resonance manipulation of qubit should be performed for the time $t\le 5\pi$. On the other hand, for the relatively larger $\alpha$ value the time point of zero density disappears. The driving frequency $\Omega =5$ in Fig. 2(d) further leaves from the resonance one that results in the distribution width decreases to $|x_c|\approx 3$. And the larger SOC intensity $\alpha = 2$ means no zero density appearing at any time. For the same $\alpha$ and $\Omega$ values, we make the spatiotemporal evolution images of all density components, and most of them are not displayed in the paper. All the results consistently prove that different density components possess a similar distribution envelope, but exist distinguishable difference of the distribution detail such that they have different zero density times for a minor $\alpha$ value. *The spatial sizes of the density components depend on whether the driving frequency nears the resonance one, while increasing the SOC intensity value can avoid appearance of the zero density component*. Notice that the phase of any state in Eq. (14) is an aperiodic function of time, because of the time-dependent phase factors in Eq. (14) being proportional to $\alpha ^2t$ and *gt*. However, Fig. 2 shows that in the case $\Omega \ne 1$, any probability density periodically oscillates with the same time period *T* adjusted by the system parameters. *These properties can be used to implement a two-qubit phase gate*^40^, by selecting the operation times $t= kT$ for any integer *k* to turn off the ac driving for purposively changing only the phases of each spin state.

### Controlling quantum transfers among different spin states

Taking $n=m$ in Eq. (15) gives the interesting relation

21 $$\begin{aligned} P=\sum _{i,j=1}^{2}P_{\eta _i\eta _j,nn'}(t)=1,\ \ P_{\eta _i\eta _j,nn'}(t)=\int _{-\infty }^{\infty }\int _0^{\infty }| \psi _{\eta _i\eta _j,nn'}(x_c,x_r,t)|^2dx_c dx_r \end{aligned}$$

between the time-independent total probability *P* and the time-dependent probability components $P_{\eta _i\eta _j,nn'}(t)$ of the particles being in the spin states $|\eta _i\eta _j \rangle$. Time evolutions of the probability components describe quantum transfers among different spin states. The phase coherence of $|\psi _{\eta _i\eta _j,01}|^2$ can be employed to control the state transfers for designing a two-qubit quantum gates.

#### Effects of magnetic field on the state transfer rates

**Figure 3:**
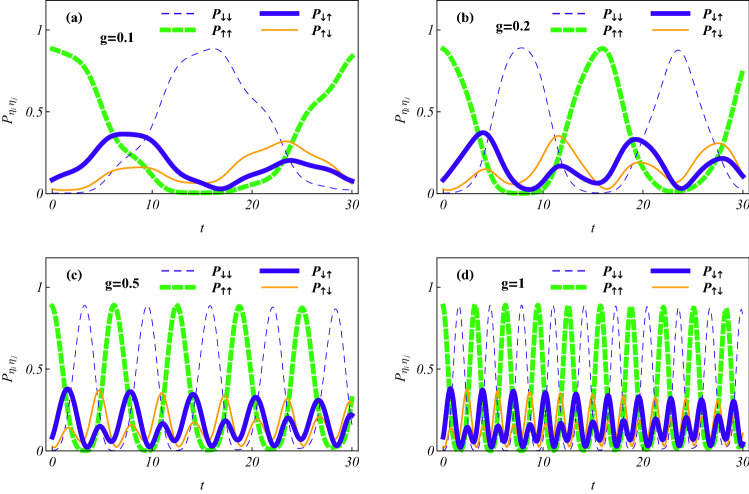
Time evolutions of the probabilities occupying spin states showing the effect of magnetic field on state transfer for the parameters $\zeta = 0, \Omega = 0, x_ 0 = 1, \alpha = 0.1$; the initial constant set $\{C_k\}$ of Fig. 1 and the different magnetic field strength (**a**) $g = 0.1$, (**b**) $g = 0.2$, (**c**) $g = 0.5$ and (**d**) $g =1$. The results mean that transfer rate of spin state $|\eta _i \eta _j\rangle$ associated with that of the probability $P_{\eta _i \eta _j}$ from a maximum to zero, is approximately proportional to magnetic field strength. The spin flip of each electron periodically occurs with flip time being determined by the controlled *g* value.

**Figure 4:**
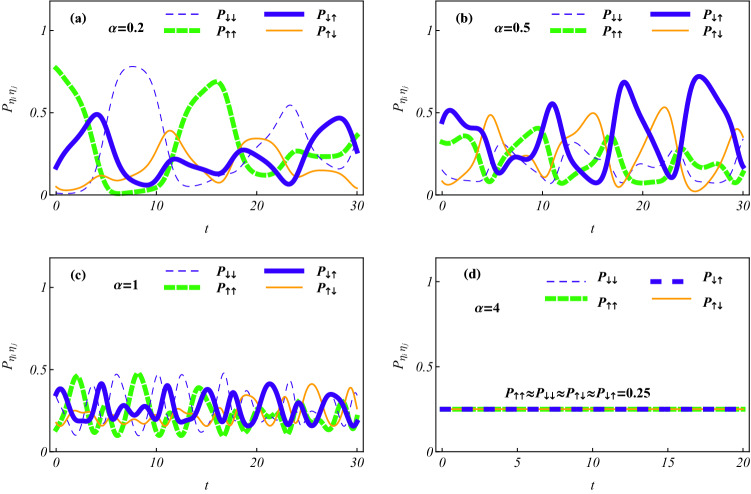
Time evolutions of the occupying probabilities showing the suppression of SOC intensity to state transfer for (**a**) $\alpha =0.2$, (**b**) $\alpha =0.5$, (**c**) $\alpha =1$, (**d**) $\alpha =4$, and the same initial constants and other parameters as those of Fig. 3(b). With the increase of $\alpha$ value, the probabilities $P_{\eta _i \eta _j}$ occupying state $|\eta _i \eta _j\rangle$ decrease their oscillation amplitudes, until to zero, meaning no transfer to occur among different spin states. The situation of approximate equal-probability appears in (d) with $\alpha =4$, which corresponds to the approximate maximally-entangled state.

In Figs. 3 and 4, the probability components $P_ {\uparrow \uparrow , 01}, P_ {\downarrow \downarrow ,01}, P_ {\uparrow \downarrow ,01}$ and $P_ {\downarrow \uparrow ,01}$ correspond, respectively, to the thick dashed, thin dashed, thin solid and thick solid curves. By Fig. 3 we demonstrate that for a smaller $\alpha$ value all the probability components periodically oscillate with zero minimum and the two maxima, $P_{\uparrow \uparrow , 01}= P_ {\downarrow \downarrow ,01}\approx 0.89$ and $P_ {\uparrow \downarrow ,01}=P_ {\downarrow \uparrow ,01}\approx 0.35$, at different time points which are determined by the controlled magnetic field strength implied in *g*. In a same time interval and for any probability component, the greater *g* value is associated with more zero probability points and higher transfer rates between spin states, which corresponds to the higher change rates of probability $P_{\eta _i \eta _j}$ from a maximum to zero with a shorter time. Taking the spin states $|\uparrow \uparrow \rangle$ and $|\downarrow \downarrow \rangle$ as examples, the state transfer times are $t_a\approx 14, t_b\approx 7, t_c\approx 3.5$ and $t_d\approx 1.75$ for (a) $g = 0.1$, (b) $g = 0.2$; (c) $g=0.5$ and (d) $g = 1$, respectively. In any case, the states $|\uparrow \uparrow \rangle$ and $|\downarrow \downarrow \rangle$ transfer each other from the probabilities $[P_ {\uparrow \uparrow , 01}(0), P_ {\downarrow \downarrow ,01}(0)] = (0.89, 0)$ to $[P_ {\uparrow \uparrow , 01}(t_k), P_ {\downarrow \downarrow ,01}(t_k)]=(0, 0.89)$, and the states $|\uparrow \downarrow \rangle$ and $|\downarrow \uparrow \rangle$ transfer each other from the probabilities $[P_ {\uparrow \downarrow , 01}(0), P_ {\downarrow \uparrow ,01}(0)] = (0, 0.11)$ to $[P_ {\uparrow \downarrow , 01}(t_k), P_ {\downarrow \uparrow ,01}(t_k)]=(0.11, 0)$ for $k=a,b,c,d$. Interestingly, such two transfers just correspond to a *spin flip of each electron* with flip time $t_k$ being the half-period of $P_{\eta _i\eta _j,01}(t)$ determined by the experimentally controllable *g* value. Thus, according to the exact solutions, we can *transparently manipulate the state transfer rates by selecting and adjusting the magnetic field strength*.

#### Suppression of SOC to the state transfer

In Fig. 3(b) we have seen that for the smaller value $\alpha =0.1$, the four probability components oscillate with minimum vanishing, and the two pair $(P_ {\uparrow \uparrow , 01}, P_ {\downarrow \downarrow , 01})$ and $(P_ {\uparrow \downarrow , 01}, P_ {\downarrow \uparrow ,01})$ have two different maxima, and the former maximum is greater than that of the latter. In Fig. 4 we further show the dependence of SOC intensity on the probabilities occupying different spin states. When $\alpha$ values are increased to 0.2 in Fig. 4(a) and 0.5 in 4(b), the former maximum decreases and the latter one increases compared to that of Fig. 3(b), until each maximum becomes different and the former maximum is less than that of the latter. In case $\alpha = 1$ of Fig. 4(c), oscillation amplitude of every probability component further decreases to obey $0< P_ {\eta _i\eta _j} < 0.5$ and tending to the approximately same one. For the larger value $\alpha = 4$ of Fig. 4(d), all the oscillation amplitudes become approximate zero and all the probabilities fall on the same value $P_{\eta _i \eta _j}\approx 1/4$ with state transfer rate vanishing. The numerical result means that the electrons being in the highly entangled superposition state of the four spin states with the approximately same probability occupying each spin state, which possesses *the approximate maximal entanglement measured by the average linear entropy*^56^.

### Manipulating mean entanglement and maximally entangled state

Clearly, applying Eq. (14) to Eq. (2) results in a set of entangled states between the two electron spins. The entanglement can be quantified by the average linear entropy^56–58^ associated with the reduced density operator $\rho _1 (t)$ on the electron 1^59^,

22 $$\begin{aligned} \rho _1 (t)& = \int \int \rho _ 1 (x_c, x_r, t) dx_c dx_r =\int \int Tr_2 \rho (x_c, x_r, t) dx_c dx_r \nonumber \\& = \int \int \sum _{j = 1}^2 [_ 2 \langle \eta _j |\psi _{01}(x_c, x_r,t) \rangle \langle \psi _{01}(x_c, x_r,t)|\eta _j\rangle _2] dx_c dx_r \nonumber \\& = \int \int \Big [(|\psi _{\uparrow \uparrow , 01}|^2+|\psi _{\uparrow \downarrow ,01}|^2)|\uparrow \rangle \langle \uparrow | +(|\psi _{\downarrow \downarrow ,01}|^2+|\psi _{\downarrow \uparrow ,01}|^2)| \downarrow \rangle \langle \downarrow | \nonumber \\& \quad +(\psi _{\uparrow \uparrow , 01}\psi ^*_{\downarrow \uparrow ,01} +\psi _{\uparrow \downarrow ,01}\psi ^*_{\downarrow \downarrow ,01}) |\uparrow \rangle \langle \downarrow |+(\psi _{\downarrow \uparrow ,01} \psi ^*_{\uparrow \uparrow , 01}+\psi _{\downarrow \downarrow ,01} \psi ^*_{\uparrow \downarrow ,01})|\downarrow \rangle \langle \uparrow |\Big ] dx_c dx_r \nonumber \\& = \left( \begin{array}{cc} P_{\uparrow \uparrow ,01}+P_{\uparrow \downarrow ,01}\ \ \ \ \ Q^*_{11}+Q^*_{12} \\ \ \ \ Q_{11}+Q_{12}\ \ \ \ \ \ P_{\downarrow \downarrow ,01}+P_{\downarrow \uparrow ,01} \end{array}\right) , \nonumber \\ Q_{11}& = \int \int (\psi _{\uparrow \uparrow , 01} \psi ^*_{\downarrow \uparrow ,01}) dx_c dx_r, \nonumber \\ Q_{12}& = \int \int (\psi _{\uparrow \downarrow ,01}\psi ^*_{\downarrow \downarrow ,01}) dx_c dx_r. \end{aligned}$$

Here, “$Q^*$” and “$\psi ^*$” denote the conjugate complex quantities of *Q* and $\psi$. Given Eq. (21), as the entanglement measure the linear entropy and the average linear entropy are defined as^56^

23 $$\begin{aligned} L(\rho _1, t)& = 1-Tr[\rho ^2_ 1 (t)]=1-[(P_{\uparrow \uparrow ,01} +P_{\uparrow \downarrow ,01})^2+(P_{\downarrow \downarrow ,01} +P_{\downarrow \uparrow ,01})^2+2|Q_{11}+Q_{12}|^2], \nonumber \\ L_A& = \frac{1}{\Delta T} \int _ 0^{\Delta T} L (\rho _1, t) dt. \end{aligned}$$

Here $\Delta T$ is a long-enough time interval such that the average linear entropy $L_A$ is insensitive to its value.

**Figure 5:**
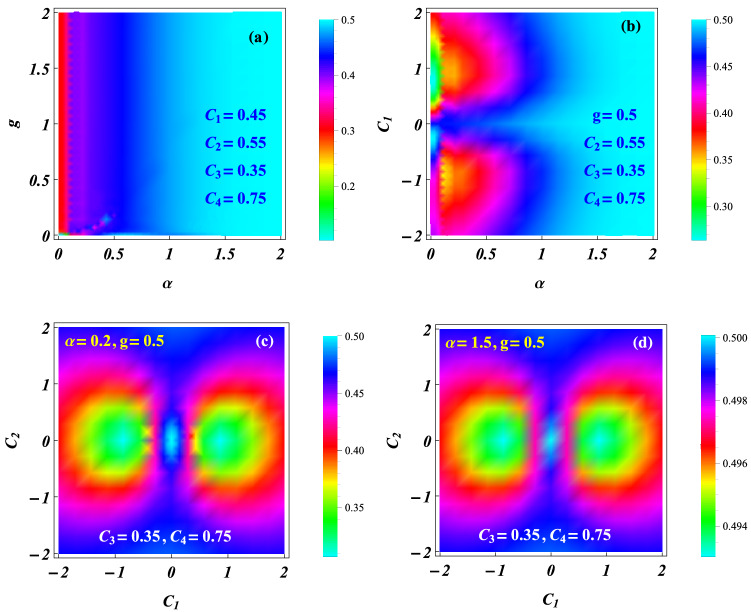
Average linear entropy $L_A$ showing the mean entanglement as functions of some system parameters and initial constants. We consider the case $f_{n_k} (x_c, t) = f_0 (x_c, t)$ and $\Delta T = 100, b_1 = 0, C_3 = 0.35, C_4 = 0.75$ for (**a**) $L_A$ vs $(\alpha , g)$ with $C_1 = 0.45, C_2 = 0.55$; (**b**) $L_A$ vs $(\alpha , C_1)$ with $g = 0.5, C_2 = 0.55$; (**c**) $L_A$ vs $(C_1, C_2)$ with $\alpha = 0.2, g = 0.5$; and (**d**) $L_A$ vs $(C_ 1, C_2)$ with $\alpha = 1.5, g =0.5$. Values of $L_A$ are indicated by the corresponding colour-number correspondence images. The results show that the mean entanglement is adjusted by the SOC intensity and the initial constants. Under the given conditions, all the average line entropies are greater than zero and increase with SOC intensity to approach its maximum $L_A= 0.5$ for $\alpha > 1.5$. Wider areas associated with the approximate maximal entanglement in (a) and (b) mean the insensitivity of the maximal entanglement to the parametric and initial perturbations.

For the case $n_k=0$, $f_{n_k} (x_c, t)=f_0 (x_c,t)$ becomes a common factor of all the motional states and the affect of its auxiliary function $b_ 1 (t)$ to the mean entanglement of state (2) is negligible. In such a case, based on Eq. (22) and considering the parameters $\Delta T = 100, b_1 = 0, C_3 = 0.35,C_4 = 0.75$, we numerically display the average linear entropy as different functions of some system parameters and initial constants, as shown in Fig. 5, where values of $L_A$ are indicated by the corresponding colour-number correspondence images with the maximum 0.5. The $\alpha -g$ plan image is exhibited in Fig. 5(a) for the constants $(C_ 1, C_ 2) = (0.45, 0.55)$. Clearly, for any fixed *g* value, the average linear entropy increases with enlarging $\alpha$ value, while for any fixed $\alpha$ value, $L_A$ is almost a constant. In Fig. 5(b) with variable $C_1$ and the same constants as those of (a) and the fixed value $g=0.5$, the image of $L_A$ vs $(\alpha ,C_1)$ displays that for any fixed $C_1$ value, the effect of $\alpha$ on $L_A$ is similar to that of (a). The approximate symmetry on $C_1 =0$ means that $L_A$ depends roughly on the absolute value of $C_1$. The different initial constants determine the corresponding motional states of Eq. (14). In Fig. 5(c,d), we investigate the average linear entropy as a function of the initial constants $(C_ 1, C_ 2)$ for $g = 0.5$, $\alpha =0.2$ in (c) and $\alpha =1.5$ in (d). We show that in (c) the different states distinguished by $C_1, C_2$ values possess distinguishable mean entanglements for the small value $\alpha = 0.2$, as indicated by the colour-number correspondence images of the right hand side. The larger value $\alpha =1.5$ in (d) makes the average linear entropy to approach the maximum $L_A=0.5$, since the colour-number correspondence image of (d) exhibits the minimal number $L_A \approx 0.493$ in this case. Any point on the images of (c) and (d) is associated with a set of fixed initial constants which determines a single ground state. Therefore, Fig. 5(d) means that all the ground states corresponding to all $(C_ 1, C_ 2)$ points have the approximately maximal mean entanglement for the larger SOC intensity $\alpha =1.5$. The approximate maximal entanglement is shown in Fig. 5(a,b) for the wider regions $(g>0, \alpha >1.5)$ and $(|C_1|\ge 0, \alpha >1.5)$, respectively. The wider areas associated with the maximal entanglement mean its insensitivity to the parametric and initial perturbations. In fact, in such regions, the effect of the small changes to the system parameters and initial constants on the mean entanglement is negligible. The result is in agreement with that of Fig. 4d. We also draw numerically the mean entanglement images for different $(C_3, C_4)$ values and the results similar to those of Fig. 5 are found. All the results consistently display that the stronger SCO makes the exact ground state of Eq. (2) the maximally entangled state with the perturbation-insensitive maximal entanglement. *Applying such maximally entangled states to encode qubits for the quantum information processing can render the qubit control more transparent and robust*.

### A new resonance transition mechanism and transparent quantum-state manipulations

In quantum mechanics, it is well-known for us to create a transition from an initial state to a desired final state by using an ac field with resonance frequency matching the level difference between the two states. However, the usual quantum transition depends only on the frequency match condition but is independent of amplitude of the ac field^59^. Later, the anomalous multiphoton-transition was found^60^, which depends only on the amplitude of the ac field, but does not relate to the frequency match condition. In both the usual and anomalous transition processes, time evolutions of the expected energy are unclear such that the transfer time to the final state is controversial. In this subsection, we will demonstrate a new resonance transition mechanism in which the quantum transition is controlled by the amplitude of the ac field. The level differences between the initial and final states are some integer times of the driving frequency $(\hbar =1)$, and the ladder-like time-evolution of the expected energy is exactly described during the transition process. Consequently, *we can transparently manipulate transitions between the exact quantum states with a high precision*.

**Figure 6:**
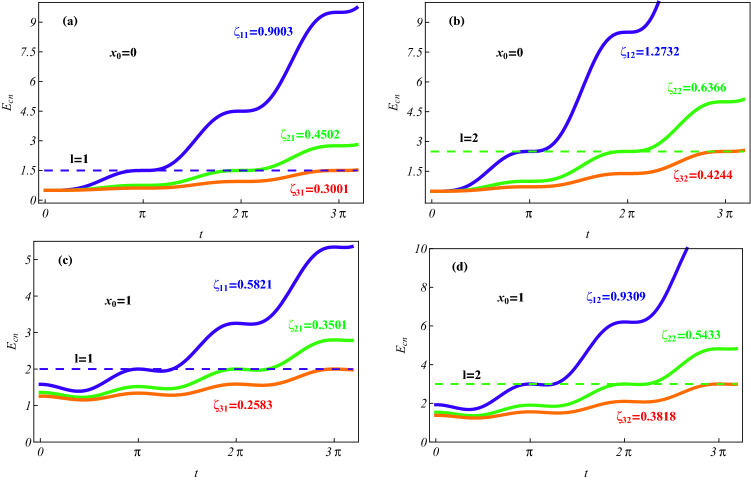
Time evolutions of the expected energy $E_{cn} (t)$ showing the resonance transition processes from initial state $|\psi _{nn'} (x_c, x_r, 0)\rangle$ with $n = 0, \zeta = \zeta _ {kl}, \Omega = 1$ and an arbitrary $n'$ to the desired *l* state $|\psi _{ln'} (x_c, x_r, t_k)\rangle$ with $\zeta =0$ at time $t_k = k\pi$ for $k = 1$ (blue), $k =2$ (green) and $k =3$ (red). The constant $x_0$ and control parameter $\zeta _{kl}$ are taken as (**a**) $x_0=0, l = 1, \zeta _{11} = 0.9003, \zeta _{21} = 0.4502, \zeta _{31} =0.3001$; (**b**) $x_0 = 0, l = 2, \zeta _{12} = 1.2732, \zeta _{22}= 0.6366, \zeta _{32} =0.4244$; (**c**) $x_0 = 1, l = 1, \zeta _{11} = 0.5821, \zeta _{21} = 0.3501, \zeta _{31} =0.2583$; (**d**) $x_0 = 1, l = 2, \zeta _{12} = 0.9309, \zeta _{22} =0.5433; \zeta _{32} =0.3818$. The dashed lines indicate the level $E_{cl} (\zeta =0)= \frac{1}{2} + l + \frac{1}{2} x_ 0^2$. As shown in this figure, the resonance transition from the ground state to any *l* state with transition time $t_k = k\pi$ is transparently controlled by selecting the ac field strength $\zeta =\zeta _{kl}$.

To investigate the new transition mechanism, we firstly prove that for the resonance frequency $\Omega =1$ time evolution curve of the expected energy exists ladders with the centre point $t =t_k=k\pi$ obeying ${\dot{E}}_{cn} (t_k) =0$ for $k=0, 1, 2, \ldots$. From Eqs. (16) and (20) we have ${\dot{E}}_{cn} (t) = {\dot{b}}_ 1[\ddot{b}_1 +b_1 + \zeta \cos (\Omega t)] + \zeta \sin (\Omega t) b_ 1 = \zeta \sin (\Omega t) b_1$ with $b_1$ obeying the driven classical harmonic oscillator equation^49^ $\ddot{b}_ 1 + b_ 1 + \zeta \cos (\Omega t) =0$. The result implies ${\dot{E}}_{cn} (\zeta =0)= 0, \ E_{cn} (\zeta =0)=$ constants or ${\dot{E}}_{cn} (\zeta \ne 0,\ t_k) = 0, \ E_{cn} (t_k)=$ constants for $t = t_k = k\pi /\Omega , \ k = 0, 1, 2, \ldots$ and any $\Omega$ value. The resonance case means $t_k = k\pi$ and the dependence of $E_ {cn} (t_k)$ on $t_k^2$. In fact, by substituting the resonance frequency $\Omega =1$ into Eq. (18), or directly solving above harmonic oscillator equation with $\Omega =1$ and for the initial conditions $b_ 1 (0) = x_0, {\dot{b}}_1 (0) =0$, we obtain the solution $b_1(t)|_{\Omega =1} = \frac{\zeta }{2}[\cos t \sin ^2 t - \sin t (t + \sin t \cos t)]+x_0\cos t$, where the resonant effect is described by the term $t\sin t$. Then from Eq. (16) we derive $E_ {cn} (\zeta \ne 0, t_k)|_{\Omega =1} = \frac{1}{2} + n + \frac{1}{2} x_ 0^2 + \zeta x_ 0 + \frac{\pi ^2}{8}\zeta ^2 k^2$. If we turn on the ac field at $t= 0$ then turn off it at $t_k =k \pi$, the energy can evolve from a initial *n* level $E_{cn}(\zeta =0, 0) = \frac{1}{2} + n + \frac{1}{2} x_ 0^2$ to the final level $E_{cn} (\zeta \ne 0, t_k)$. In order to realize the transition to the desired *l* level, we must select an appropriate $\zeta$ value to obey $E_{cn} (\zeta \ne 0, t_k)=E_{cl} (\zeta = 0) = \frac{1}{2} + l + \frac{1}{2} x_ 0^2$, namely the ac field strength should be selected to satisfy the equation $\zeta x_ 0 + \frac{\pi ^2}{8}\zeta ^2 k^2 =l - n$ with the solution

24 $$\begin{aligned} \zeta =\zeta _ {kl} = \frac{4}{k^2\pi ^2}\Big [-x_ 0 + \sqrt{x_ 0^2 + \frac{k^2\pi ^2}{2} (l - n)}\Big ] \end{aligned}$$

for the initially given constants $x_0\ge 0$ and $n<l$. In Fig. 6 we illustrate that application of the driving strength $\zeta _{kl}$ leads to the transition from any initial *n* state $|\psi _{nn'}(x_c, x_r, 0)\rangle$ with $\zeta = \zeta _{kl}$ to the desired *l* state $|\psi _{ln'}(x_c, x_r, t)\rangle$ with $\zeta =0$ for the determined time $t=t_k = k\pi$. However, as an inverse of the time units the frequency exists a certain width such that an infinitely accurate $t_k$ value is impossible to experimentally set. Thus we have to consider the transition time $t_ {kf}$ being in a time interval $\Delta t$ centred at $t_k$, which is associated with a small level width $\Delta E$. To realize a transition with high precision, such a time interval should correspond to a small ladder width of energy curves.

The time-dependent energy $E_{cn} (t)$ is independent of the initial constant set $\{C_k\}$ and system parameters $\alpha , g$. We take the initial ground state with $n =0$ as an example without loss of generality. In Fig. 6, we plot the time evolutions of the expected energy in the resonance case, which *show the transition process from an initial ground state to the desired* *l* *excitation state*. We find that the centres of energy plateaus appear at $t_k =k\pi$ and all the ladders have an approximately same width $\Delta t\approx \frac{\pi }{4}$ for the transition time $t_ {kf} \in [t_k - \pi /8, t_k + \pi /8]$. The corresponding level width $\Delta E =max | E_ {cn} (t_k) - E_ {cn} (t_ {kf})|$ is in order of $10^{-2}$. It is worth noting that when the transition is finished by turning off the ac field at $t = t_{kf}$, the minor level width results in the energy-time uncertainty relation $\Delta E \Delta t \ll 1 (\hbar )$, meaning a quite high operation precision. While the greater ladder width $\Delta t \approx \frac{\pi }{4}$ leads to the transition times $t_ {kf}$ being in the experimentally appropriate interval for an usual frequency width $\Delta \omega \ll 1 (\omega )$. In addition, by comparing the different energy curves, we find that the larger *k* value relates to the smaller driving strength. The result implies that a weak ac field also can cause the level transition after a longer time, and for a fixed *l* final state the shortest transition time $t_{kf}$ with $k =1$ is associated with the highest driving strength $\zeta _{1l}$.

## Summary

In summary, we have investigated two SO coupled electrons held in a quantum-dot hybrid 1D nanowire^27^, subject to an ac electric field and a static magnetic field, which is governed by the effective Hamiltonian in Eq. (1). By managing the orientation of the static magnetic field to match the SOC-dependent phase^12^ and selecting the specific trapping frequencies to fit the exact stationary states of relative motion experiencing the Coulomb interaction and the harmonic potential simultaneously^44–47^, we have acquired a set of exact orthonormalized spin entangled states of Eq. (2) with probability amplitudes being the motional states. Combining the function-transformation method and the variable-separation treatment, exact complete solutions of the motional states have been constructed in Eq. (14) as the coherent superpositions of the known generalized coherent states with some arbitrary constants determined by the initial states. The square norm of a motional state describes the probability density occupying the corresponding spin state and behaves as a kind of oscillating wave packets. The different initial constant sets can correspond to the different ground states with the same lowest quantum number and the same or different expected energies. For any ground state, the spatiotemporal evolutions of the probability densities can be adjusted by the ac electric field and the intensities of SOC and magnetic field, as shown in Figs. 1 and 2, where the shapes and sizes of the density wavepackets and the numbers, locations and height of the wave peaks depend mainly on SOC intensity and driving frequency. In Figs. 3 and 4, the time evolutions of probabilities occupying different spin states reveal that transfer rate between the spin states is approximately proportional to magnetic field strength for a weaker SOC, and the transfer can be effectively suppressed by enhancing the SOC intensity. The effects of the system parameters and initial constants on the mean entanglements measured by the average linear entropy have been illustrated numerically by Fig. 5, where the approximately maximal mean entanglement is associated with the stronger SOC and wider regions of the system parameters and initial constants, meaning the insensitivity to the parametric and initial perturbations. In any one of the orthonormalized states of Eq. (2), the expected energy of Eq. (16) contains a quantum part and a continuously time-varying one. Applying the frequency resonance effect, by Fig. 6 and Eq. (23) we have demonstrated a novel resonance transition mechanism in which the ladder-like time evolution of expected energy and the corresponding transition time between two arbitrary states are transparently controlled by the ac field strength implying in the exact motional states. The exact ground states with the perturbation-insensitive maximal entanglement can be used to encode qubits and to render the qubit control more transparent and robust.

Treating the exact solutions as leading-order ones, the obtained results could be applied to the locally gated few-dot system or an array of electron pairs separated from each other by different quantum dots with weak neighboring coupling as perturbation. The latter may have practical importance to scale up quantum computation with quantum-dot-electron system. Our results also show the coherent control of qubits in low-dimensional electronic systems, which is fundamental important to design of solid-state quantum circuits and for encoding spin qubits via the maximally entangled ground state. In the further work, applying the theoretical proposal of geometric gates with the reduced sensitivity to the vibrational quantum numbers^61,62^, we will implement the two-qubit phase gates by using the state-dependent forces to manipulate the exact states^38,40^. We will also extend the exact results to a 2D two-electron quantum-dot system^28,46^.
